# Robust detection of periodic time series measured from biological systems

**DOI:** 10.1186/1471-2105-6-117

**Published:** 2005-05-13

**Authors:** Miika Ahdesmäki, Harri Lähdesmäki, Ron Pearson, Heikki Huttunen, Olli Yli-Harja

**Affiliations:** 1 Institute of Signal Processing, Tampere University of Technology, P.O. Box 553, 33101 Tampere, Finland; 2 ProSanos Corporation, Harrisburg PA 17101, USA

## Abstract

**Background:**

Periodic phenomena are widespread in biology. The problem of finding periodicity in biological time series can be viewed as a multiple hypothesis testing of the spectral content of a given time series. The exact noise characteristics are unknown in many bioinformatics applications. Furthermore, the observed time series can exhibit other non-idealities, such as outliers, short length and distortion from the original wave form. Hence, the computational methods should preferably be robust against such anomalies in the data.

**Results:**

We propose a general-purpose robust testing procedure for finding periodic sequences in multiple time series data. The proposed method is based on a robust spectral estimator which is incorporated into the hypothesis testing framework using a so-called *g*-statistic together with correction for multiple testing. This results in a robust testing procedure which is insensitive to heavy contamination of outliers, missing-values, short time series, nonlinear distortions, and is completely insensitive to any monotone nonlinear distortions. The performance of the methods is evaluated by performing extensive simulations. In addition, we compare the proposed method with another recent statistical signal detection estimator that uses Fisher's test, based on the Gaussian noise assumption. The results demonstrate that the proposed robust method provides remarkably better robustness properties. Moreover, the performance of the proposed method is preferable also in the standard Gaussian case. We validate the performance of the proposed method on real data on which the method performs very favorably.

**Conclusion:**

As the time series measured from biological systems are usually short and prone to contain different kinds of non-idealities, we are very optimistic about the multitude of possible applications for our proposed robust statistical periodicity detection method.

**Availability:**

The presented methods have been implemented in Matlab and in R. Codes are available on request. Supplementary material is available at: .

## Background

Periodic phenomena are widespread in biology, including, among others, membrane potential oscillations, cardiac rhythms, smooth muscle contraction, calcium oscillations, protoplasmic streaming, glycolytic oscillations, cAMP oscillations, oscillations in neuronal signals, insulin secretion (pancreas), gonadotropic hormone secretion, cell cycle, circadian rhythms, and ovarian cycle (see e.g. [1]). Consequently, there are numerous biological applications where periodicities must be detected from experimental biological data. Because the data measured from biological applications are inherently noisy and usually sparsely sampled, efficient algorithms are being developed to extract as much information as possible.

In the past few years there has been an explosion of available microarray gene expression data. Detecting periodicity in gene expression is of great importance because it indicates, e.g., cell-cycle regulation [2] as well as the effect of circadian rhythms [3]. The significance of the detection of cell-cycle regulated processes is further emphasised by the linkage between cell-cycle and cancer (see, e.g., [4] and [5]). To this end, microarrays have been used to study the circadian gene expression in *Neurospora Crassa *[3] as well as cell-cycle regulated genes, e.g., in budding yeast [6], in fission yeast [7], and in human cells [5].

The task of finding periodicity in time series measured from a biological system can be viewed as a decision problem based on spectral analysis together with multiple hypothesis testing. A formal statistical testing procedure for the detection of periodic expression profiles was recently introduced by Wichert *et al. *[8]. It relies on the use of a so-called Fisher's *g*-statistic for which the exact null-distribution can be derived under the Gaussian noise assumption.

Recently, a number of other methods for detecting periodic transcripts have also been proposed [9-13]. A major difference between the method proposed by Wichert *et al. *[8] and other methods is that Wichert's method is capable of detecting unknown frequencies whereas other methods are designed for detecting fixed frequencies. From a computational point of view, the problem of finding unknown frequencies is even more demanding since no prior knowledge of the frequency to be detected is available. In many biological applications it is more important to search for periodicities having an unknown frequency. However, in some applications, such as large-scale cell cycle studies, the period length is usually known and thus provides additional information for testing. In this paper, our goal is to tackle both of the two problems. That is, we develop two methods, one for the detection of unknown frequencies and the other for testing fixed frequencies.

In many applications, including those arising from bioinformatics, the exact noise characteristics are usually unknown and can be remarkably non-Gaussian. Furthermore, the observed gene expression time series can exhibit other non-idealities, such as outliers, short length and distortion from the original wave form. Therefore, it would be useful to have a robust method for detecting periodic components, i.e., a method that also works well when the original (Gaussian) noise assumption no longer holds.

A robust, rank-based, non-parametric spectral estimator was recently introduced in [14]. In this paper, we extend the approach of [14] to the detection of periodic time series. This results in a robust testing procedure which is insensitive to a heavy contamination of outliers, missing-values, short time series, nonlinear distortions, and is completely insensitive to any monotone nonlinear distortions. We also consider a permutation-based alternative to the method proposed in [8] and show that, when the data is contaminated with the above mentioned non-idealities, this results in a more robust method.

As discussed, e.g., in [15], the performance of a method can be proven using either extensive simulations, analytical proofs or multiple plasmode experiments (to use a term from [15]). Plasmode is a real data structure whose true structure is known. Although very useful, the kinds of benchmarkings proposed in the literature (related to periodically behaving gene expression time series) so far do not belong to any of the above categories. In particular, the proposed benchmarking frameworks cannot be considered as plasmodes since the true structure (periodic genes) is not known but, instead, is based on partial biological knowledge (or other measurements, such as protein-DNA binding). Hence, the performance cannot be assessed solely on real data. Therefore, as the analytical proofs are hard to obtain in this case, we perform extensive simulations to show the superior performance of the proposed methods. However, we also apply the proposed method to real experimental data to show that the methods perform well on real data and that the results are biologically meaningful.

## Results

### Computational methods

In order to be consistent with the previously published methods, we use similar notation as in [8] and also consider the same model for the periodic time series

*y*_*n *_= *β *cos(*ωn *+ *φ*) + *ε*_*n*_,     (1)

where *β *≥ 0, *ω *∈ (0, *π*), *n *= 1,..., *N*, *φ *∈ (-*π*, *π*], and *ε*_*n *_is an i.i.d. noise sequence. To test for the periodicity, define the null hypothesis as *H*_0 _: *β *= 0, i.e., time series consists of the noise sequence alone, *y*_*n *_= *ε*_*n*_. In the following we develop a method for detecting unknown frequencies and later introduce a modification which can be applied to the detection of known frequencies. We first review the Fisher's test for the detection of periodic transcripts as introduced by Wichert *et al. *in [8].

#### Wichert's method

The method proposed by Wichert *et al. *[8] is based on the periodogram spectral estimator, defined as



where *N *is the time series length. The periodogram is further evaluated at (harmonic) normalised frequencies



where *a *= [(*N *– 1)/2] and [*x*] denotes the integer part of *x*. To test for the periodicity formally, some kind of a test statistic must be chosen. The so-called *g*-statistic for one time series is given by



In plain words, the *g*-statistic is the maximum periodogram ordinate divided by the sum of all periodogram ordinates for *l *= 1, ..., *a*. Large value of *g *indicates a strong periodic component and leads to the rejection of the null hypothesis.

Wichert *et al. *[8] resort to a result by Fisher that, under the Gaussian noise assumption, gives the exact distribution of the *g*-statistic under the null hypothesis (see, e.g., [16,8]). The exact *p*-value for a realisation of the *g*-statistic is shown to be



where *b *is the largest integer less than 1/*x *and *x *is the observed value of the *g*-statistic. Because there are usually thousands of time series that are tested simultaneously, whether they exhibit periodicity or not, there is a possibility that a time series can have a small *p*-value by chance. To correct the *p*-values for multiple testing, Wichert *et al. *[8] use the method of Benjamini and Hochberg (see, e.g., [17]), which controls the False Discovery Rate (FDR). The FDR method controls the expected proportion of false positives (Type I errors) at a given rate *q*. The threshold of the FDR depends on the evaluated p-values. The FDR procedure for the ordered set of *p*-values *p*_(1)_, *p*_(2)_,...,*p*_(*M*)_, where *M *is the number of time series, is as follows

1. Let *i*_*q *_be the largest *i *for which 

2. Reject the null hypothesis for the time series corresponding to the *p*-values *p*_(1)_, *p*_(2)_,..., 

The procedure based on the periodogram spectral estimator for periodicity detection has several well-known and important properties. For example, if the noise sequence ∈_*n *_is i.i.d. Gaussian and the true underlying frequencies are among the harmonic frequencies Ω = {*ω*_*l *_: *l *= 0,..., [(*N *- 1)/2]}, then the (square root of the) periodogram is the minimum variance unbiased estimator of the frequency content at discrete frequencies Ω (see, e.g., [18]). However, it is also widely recognized that the standard periodogram is an inconsistent spectrum estimator (see, e.g. [19]). Despite this weakness, the periodogram is a standard method that is theoretically well-founded. Under the popular Gaussian working assumption, widely invoked in spectral estimation, the distributional characteristics of the periodogram are known and useful. Indeed, it is this distributional characterisation that forms the basis for Fisher's *g*-test for whiteness. In other words, Equation (5) provides the exact significance value for a realisation of the *g*-statistic. This provides a solid theoretical basis for the use of the method proposed in [8].

Concerning the problem of detecting hidden periodicities, some generalisations and improvements over the traditional methods have been proposed. A recent review of the proposed methods can be found in [20]. Many of the improved methods are some type of generalisations of the traditional methods, such as the correlogram or periodogram. Artis *et al. *[20] report that two particular methods are generally found to have a good performance: so-called mixed spectrum methods by Priestley and Bhansali (see, e.g. [19,20]), and a modified method based on the maximum periodogram ordinate by Chiu [21]. The method by Priestley and Bhansali uses a certain type of windowing of the correlogram for the "smoothing" purposes (i.e., in order to reduce the variance). The method by Chiu in turn modifies the *g*-statistic by replacing the average spectrum in the denominator with a proper trimmed mean of the ranked periodogram ordinates. As we explain in the Discussion Section, the same modifications/improvements can be used in the proposed robust method (to be introduced shortly) as well. Indeed, the windowing can be utilized much in the same way as it is used in the method by Priestley and Bhansali. Similarly, the modification(s) of Chiu can be directly implemented in the case of the proposed robust estimator.

Fisher's test and the proposed robust method can be considered to be analogous in the sense that neither of them incorporates any modifications for the traditional periodogram/correlogram approach. In Simulations Section we perform an extensive comparison of the unmodified, standard methods since this provides a better insight into the performance differences of the traditional methods and the proposed robust rank-based methodology. Furthermore, from an extensive simulation point of view, comparison of many different modifications together with the robust method would also result in a rather large number of simulations, especially because it is possible to use many different combinations of the modifications. Note, however, that the further modifications, such as the ones proposed by Priestly, Bhansali, and Chiu, can be implemented in both of the frameworks (the traditional and the robust rank-based) to gain further performance improvements. We discuss this issue further in Discussion Section.

In many applications, particularly in bioinformatics, the noise distributions are usually unknown and can remarkably deviate from the Gaussian assumption. The methods based on Gaussian noise assumption may fail, or even produce invalid results, when the model assumptions do not hold. The goal of this paper is to introduce an alternative for the standard methods, aimed at providing a robust estimator.

#### A robust alternative

As a starting point we should like to remind that the periodogram *I*(*ω*) is equal equivalent to the correlogram spectral estimator (see, e.g., [25])



where  is the biased estimator of the autocorrelation function



Note that the required values for  for *m *< 0 are obtained by invoking the inherent symmetry of the autocorrelation function: *r*(-*m*) = *r*(*m*). Consequently, the *g*-statistic in Equation (4) as well as the corresponding significance value in Equation (5) can also be computed using *S*(*ω*) instead of *I*(*ω*). It is natural to try to obtain robustness by replacing  with a robust alternative.

Before continuing to the robust method, it is important to note that, especially in the case of gene expression time series, the data is often contaminated with missing values. Therefore, the spectral estimation method must take missing samples into account. Let *I*_*m *_be the set of time indices *k *for which both *y*_*k *_and *y*_*k*+*m *_are available and *K*_*m *_= |*I*_*m*_|. As long as *K*_*m *_≠ 0, a missing data-adapted version of the unbiased estimate of the autocorrelation can be obtained as



We cover only the versions adapted to missing data in the following text since they are equal to the standard estimators in case of complete data sets. Note that *K*_*m *_equals *N *- *m *for the complete data sets. Next we consider a recently introduced rank-based autocorrelation estimator [14] for the problems of spectrum estimation. This estimator is a moving-window extension of the Spearman rank correlation coefficient, quantifying the association between the sequences {*y*_*k*_} and {*y*_*k*+*m*_}. The resulting quantity, *ρ*^*S*^(*m*) is actually an alternative estimator of the standard correlation coefficient *ρ*(*m*) between these sequences (see e.g. [19])



where [·] denotes the expectation operator and *μ*_*yi *_=  [*y*_*i*_] is the mean of the sequence. Recall that the sample correlation coefficient between two *N *length sequences {*x*_*i*_} and {y_*i*_} is defined as



where  denotes the sample mean of {*x*_*i*_}.

Under the assumption of stationarity, it immediately follows from Equation (9) that the correlation coefficient *ρ*(*m*) is related to the autocorrelation function *r*(*m*) by , where  is the variance of the sequence. Since it is important to remove the mean of the sequence prior to spectrum estimation to avoid low frequency artifacts and since  is simply a scale factor, the problem of detecting periodic components in a data sequence may equally well be based on *ρ*(*m*) as *r*(*m*). Consequently, we consider spectral estimators of the form



where  estimates the correlation coefficient between {*y*_*k*_} and {*y*_*k*+*m*_} and *L *is the maximum lag for which the correlation coefficient is computed. More specifically, we consider the correlation coefficient between the data ranks *R*_*y*_(*i*) and , defined by



where *C *is a normalisation factor, *R*_*y*_(*i*) denotes the rank of *y*_*i *_in the set *S *= {*y*_*j *_: *j *∈ *I*_*m*_} and  denotes the rank of *y*_*i*+*m *_in the set *S' *= {*y*_*j*+*m *_: *j *∈ *I*_*m*_}. By selecting either *C *= *K*_*m *_or *C *= *N *in Equation (12) yields the unbiased or the biased estimate of the correlation coefficient between the rank sequences, respectively. Because both of these rank sequences assume every value from 1 to *K*_*m *_precisely once, their average is (*K*_*m *_+ 1)/2, independent of the data values {*y*_*n*_}, and their sample variance, when scaled by the sequence length *K*_*m*_, can be shown to be ( - 1)/12. More generally, if *C *= *K*_*m*_, and since *ρ*^*S*^(*m*) is the correlation coefficients between ranks, it is bounded by -1 ≤ *ρ*^*S*^(*m*) ≥ 1 for all *m*. In the following we shall use *L *= *N *- 2 in Equation (11) because *N *- 2 is the largest lag for which Equation (12) can be computed.

We shall use the biased estimate, i.e., *C *= *N*, here because of its connection to the equivalence between *I*(*ω*) and *S*(*ω*). Moreover, the use of the biased estimate in spectrum estimation (Equations (6) and (7)) can be interpreted as triangular weighting of the autocorrelation function estimate. Windowing is usually applied to reduce the scalloping loss effect which is the reason why some frequencies are inferior to others [25].

As in the case of the standard autocorrelation estimate, the values for *ρ*^*S*^(*m*) for *m *< 0 are obtained by symmetry: *ρ*^*S*^(-*m*) = *ρ*^*S*^(*m*). This also helps in computing Equation (11) since it reduces to



where (*x*) denotes the real part of *x*. As opposed to standard periodogram or the corresponding correlogram, the proposed robust spectral estimator is not guaranteed to be non-negative. We shall hence use the absolute value of  in the following.

#### Significance values

In the same way as Wichert *et al. *[8] do, we use the *g*-statistic and evaluate



for each time series spectral estimate. However, we do not have the luxury of resorting to an exact distribution of the *g*-statistic, e.g., under the Gaussian noise assumption. To obtain the significance values we consider two common ways of computing them: simulation and permutation-based methods. This also opens up a possibility for adjusting some parameters in the proposed robust method that were previously kept fixed. In particular, we vary the number of equidistant frequencies at which the spectral estimate is evaluated and change Equation (14) accordingly by incorporating more terms in the max-operator as well as in the sum in the denominator. Instead of having a fixed set of *a *+ 1 normalised frequencies as in Equation (3), we can evaluate the spectral estimate at [(*K *- 1)/2] + 1 equidistant frequencies



Although the method is rather insensitive to the selection of *K*, we found that *K *= 2*N *generally provides a good performance. Evaluating  at more frequencies can be viewed as a smoothing or interpolation of the original discrete spectral estimate. From the implementation point of view it is worth mentioning that Equation (13) evaluated at frequencies shown in Equation (15) can be computed using the fast Fourier transform (FFT).

As was already discussed in the Background Section, in some cases one might be interested in testing fixed instead of unknown frequencies. The proposed method can naturally be adapted to that case as well. If *ω*' is the known frequency for which the spectral content is to be tested, then a modified *g*-statistic, *g*', can be used



In the following, we mainly concentrate on the use of the standard *g*-statistic for detecting unknown frequencies. However, the same methods, such as simulation and permutation based significance values, can also be applied to the modified *g*-statistic. In Experimental results Section we apply both the standard and the modified *g*-statistics to real microarray data.

When the noise satisfies the i.i.d. assumption, the decision between simulation and permutation test-based significance values is facilitated by the following observation. A statistic *T *is said to be distribution-free over a collection of distributions  if the distribution of *T *is the same for every joint distribution in . Consider the signal model shown in Equation (1) under the null hypothesis *H*_0_: *β *= 0, i.e., *y*_*n *_= *ε*_*n*_, and assume that *ε*_*n *_is again i.i.d. and has continuous distribution. It is easy to see from Equation (12) that, for any time series {*y*_*n*_}_*n *= 1,...,*N*_, the *g*-statistic depends on {*y*_*n*_}_*n *= 1,...,*N *_only through the rank sequence {*R*_*y*_(*i*)}_*i *= 1,...,*N*_, where *R*_*y*_(*i*) denotes the rank of *y*_*i *_in the original sequence. This implies that the *g*-statistic is distribution-free over the class of all joint distributions of *N *i.i.d. continuous univariate random variables (see, e.g., [22]). In other words, for each *N *and independent of the type of the noise, the *g*-statistic has exactly the same null distribution as long as the noise term satisfies the continuous i.i.d. assumption. Therefore we can choose to use either the simulated distributions (must be simulated separately for each times series sequence length) or the permutation tests, depending on the circumstances.

#### Simulation-based significance values

The simulation-based method is simple. Given the model as in Equation (1) together with some distributional assumptions for *ε*_*n*_, generate a set of *P *random time series under the null hypothesis. Evaluate the test statistic shown in Equation (14) on each of the *P *time series. Use the obtained *g*-values to compute an estimate of the distribution of the *g*-statistic under the null hypothesis. The distribution can be estimated, e.g., using kernel density estimation methods. The testing can then be performed as explained above except that the significance values are computed/integrated relative to the estimated distribution. Note that the null distribution must be estimated for each time series length separately but, due to the distribution-free property, the null distribution is independent of the noise characteristics under the i.i.d. assumption.

#### Permutation-based significance values

A more flexible way of obtaining *p*-values is to use permutation tests [23]. Although they are a relatively old concept, permutation tests have only recently become interesting in practise because of the intensity of needed computing power. The idea is simple:

1. Choose a test statistic.

2. Evaluate the test statistic on the original data.

3. Randomly permute the data and evaluate the test statistic on every permutation.

4. Estimate the distribution of the test statistic with the help of the sample generated in point 3.

5. Use the estimated distribution to get a *p*-value for the original test statistic computed in point 2.

A sequence of random variables {*X*_*n*_}, *n *= 1, 2,...,*N *is exchangeable, if the joint distribution of  is the same as that of the original sequence *X*_1_, *X*_2_,..., *X*_*N *_for all permutations *π*. Under the null hypothesis, the elements of the time series *y*_*n *_are i.i.d. and therefore exchangeable, and hence the permutation test can be applied. Alternatively, as the application of a random permutation destroys any periodic structure that is present in the original sequence, permutation tests can be used to assess how highly structured the given time point values are in the light of the chosen test statistic versus other permutations of the given sample. As the concept of permutation tests is non-parametric, they can be applied without knowing the exact distribution of the data at hand.

Instead of performing all the *N*! permutations for each time series, we have chosen to permute each of the original time series for *P *= 5000 times. As our simulations show, this seems to be quite an adequate number of iterations. The selection of *P *is always a compromise, because too high *P *makes computations too slow and too low *P *weakens the accuracy and resolution of the calculated *p*-values. For example, time series having a very periodic structure can get a *p*-values of zero due to the low value of *P*.

While we have mainly applied the permutation tests to the robust estimator, it must be noted that with the help of permutation tests the robustness of the periodogram can also be improved. As we show in Results section that if we add, e.g., some impulsive noise to the simulated data, the results when using the periodogram method as in Equation (5) are not as good as when we use permutation tests to find the *p*-values.

#### Correction for multiple testing

In order to facilitate the comparison between the proposed and previous methods, the obtained *p*-values are corrected exactly in the same way as in the method by [8].

### Simulations

We put the presented methods to a test by first going through simulated data, where the ground truth is known, and then by finding periodically behaving genes in real microarray data.

**Figure 1:**
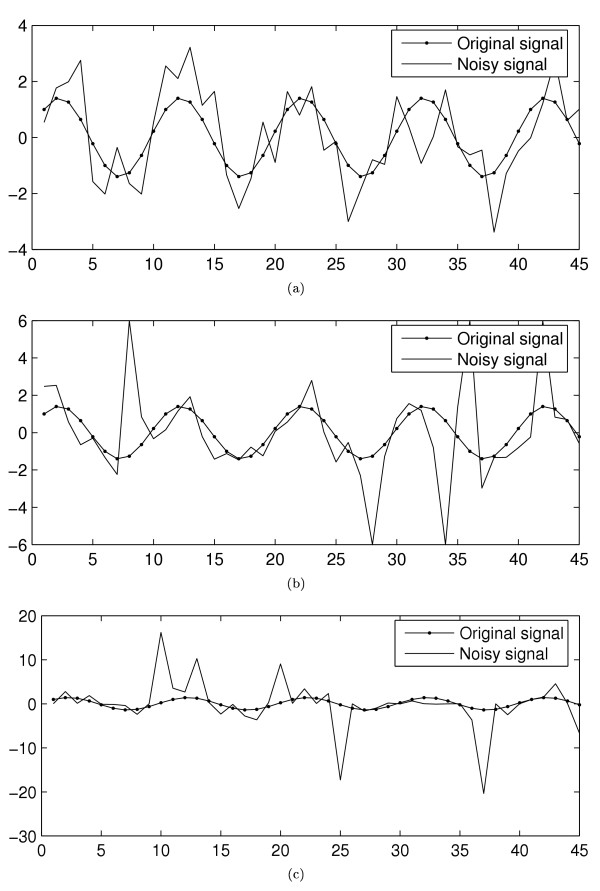
**Examples of time series**. An example of a time series composed of a sine and (a) additive standard Gaussian noise, (b) additive standard Gaussian and impulsive noise, and (c) additive standard Gaussian noise and cubic distortion.

In simulations, we use exactly the same test signal model as in [8] for comparison purposes, namely Equation (1) with *β *=  and *φ *= -*π*/4, i.e.,



where *n *= 1,...,*N*, *ω *is uniformly randomly chosen in the interval *ω *∈ [0.05*π*, 0.45*π*] (we wanted to avoid frequencies near zero and the Nyquist frequency) and *ε*_*n *_is an i.i.d. noise sequence. An essential parameter is the amplitude (*β *= ) which affects the signal-to-noise ratio and which we would like to have the same as in [8]. We chose to consider three types of non-idealities, namely (*i*) pure standard Gaussian noise (zero mean and unit variance, see Figure 1(a)), (*ii*) standard Gaussian and impulsive noise (number of impulses equals ten percent of the sequence length, amplitude ± 6 times the standard deviation of the Gaussian noise, see Figure 1(b)), and (*iii*) standard Gaussian noise and *x*^3 ^distortion, where all values were raised to the power of three after adding the noise (see Figure 1(c)).

**Figure 2:**
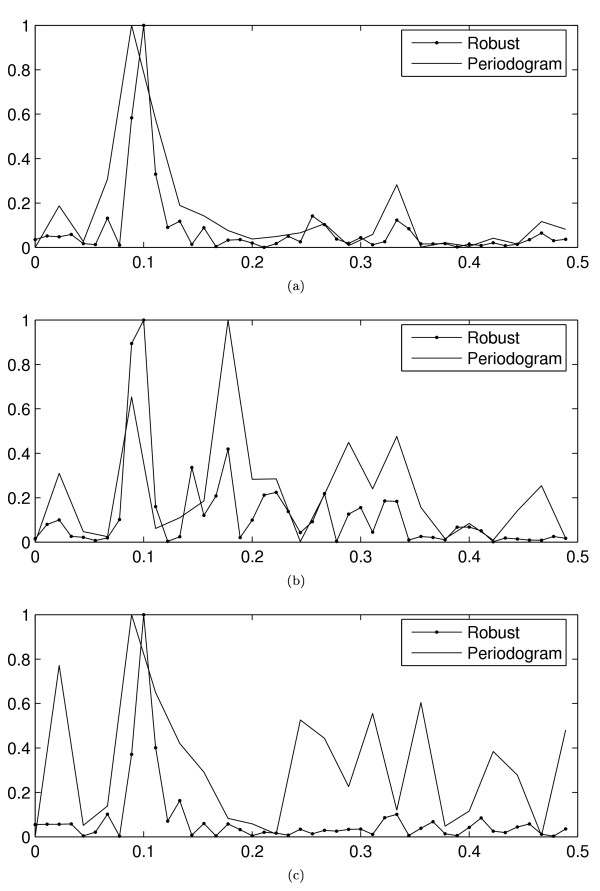
**Examples of spectral estimates**. The spectral estimates for the time series in Figures 1 (a)-(c), respectively, using both the standard periodogram and the proposed robust method.

In each example sequence in Figure 1, the normalised frequency of the original sinusoidal is *ω *= 0.1. Figures 2(a)–(c) show the spectral estimates for the time series in Figures 1(a)–(c), respectively, using both the standard periodogram and the proposed robust method. Note that the spectra have been scaled for viewing purposes.

Figures 2(b)–(c) already illustrate a remarkable difference between the two methods. For more details about the performance of the proposed robust method as a spectrum estimator, see [14]. A detailed comparison of the periodicity detection capabilities is performed next.

**Figure 3:**
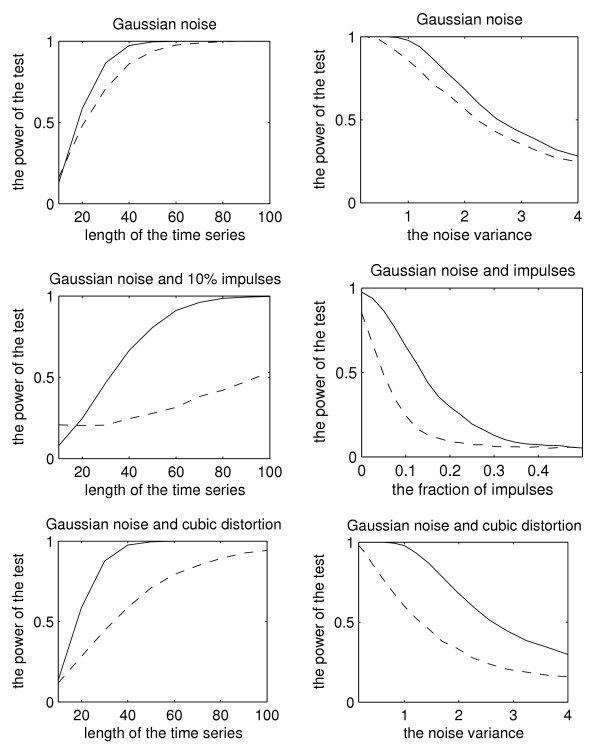
**Power of the test**. The power of the tests (*y*-axis) for the three different test cases as the function of the time series length and varying noise parameters (*x*-axis). The solid (resp. dashed) line corresponds to the proposed robust method (resp. Fisher's test). Three different types of non-idealities are considered, namely, pure standard Gaussian noise (the first row), standard Gaussian and impulsive noise (the second row), and standard Gaussian noise and *x*^3 ^distortion (the third row). The left (resp. right) column shows the results for different time series lengths (resp. different values of the noise parameters).

Let us first examine the power of the test, i.e., one minus the probability of the type II error (false negative). The power of the test is estimated for the three different test cases as well as for different time series lengths and for different noise parameters using 10000 Monte Carlo runs, see Figure 3. The significance level is set to *α *= 0.05. In all the three cases, the case-specific noise assumptions are used for both the null hypothesis (*β *= 0) and the alternative hypothesis (*β *> 0). In this simulation, we use the signal model shown in Equation (17) to represent a periodic signal (i.e., the alternative hypothesis). In the right column of Figure 3, the length of the time series is set to 40 and the power is shown as the function of varying noise parameters. Figure 3 clearly shows that the power of the proposed robust hypothesis testing method is remarkably better than that of the Fisher's test, especially in the case of outliers and non-linear distortion. More interestingly, however, the proposed method is also more powerful in the case of standard Gaussian noise.

Next we consider another simulation. In the same way as in [8], two thousand time series of length *N *= 10, 20, 40, 45, 50 and 100 were generated to test the periodicity detection. One thousand and nine hundred of the time series were plain noise and one hundred time series were generated according to Equation (17). We again consider the three aforementioned noise models. As explained in the Computational Methods Subsection, we evaluated the *g*-statistic and *p*-value for each time series and then used the FDR rule to determine which of the time series were considered to be cyclic for a certain FDR level. The FDR level, at which the expected rate of false positives is controlled, was chosen similarly as in [8], i.e., *q = *0.15, 0.10, 0.05, 0.01 and 0.005. For each *N *and *q *the simulation was run for 99 times for the simulation-based cases and 9 times for the permutation-based cases. Median statistics are reported for the number of found periodic components, the number of correctly identified periodic components (shown in parenthesis) and the number of truly periodic time series among the top 100 ranked sequences (*Z*).

If we take a look at the results in Tables 1 to 9 we can draw some immediate conclusions. First, when the noise is plain Gaussian, Tables 1, 2, 3, 4 show that both methods perform approximately equally well.

There are no significant differences between the two methods in terms of the number of detected genes or in terms of the number of correctly detected genes. However, the numbers of truly periodic genes among the top 100 ranked sequences (*Z*-scores) show somewhat favorable performance for the robust method, especially for the short time series *N *= 20 and *N *= 40. Indeed, this observation agrees with previous findings [14] where the robust method was found to have a good performance as a spectrum estimator for short time series. By comparing Tables 1 and 2 and Tables 3 and 4, it is obvious that the permutation tests do not provide any significant performance gain over the traditional approach where the significance values are computed using the simulation-based method or Equation (5), respectively. In both cases, the *Z*-scores are about the same, as expected. The only notable difference is seen in the number of found periodic genes for short time series (e.g., *N *= 40, 45, 50) and small FDR levels (*q = *0.005, 0.01, 0.05) where the numbers are slightly higher when permutation tests are used. This suggests that the permutation-based method finds a bit smaller *p*-values than the simulation-based method.

Tables 1, 2, 3, 4, 5, 6, 7, 8 clearly show the superior robustness of the proposed method over the traditional Gaussian analysis. As can be seen from Tables 1 and 5 and Tables 2 and 6, there is only a minor performance degradation between the Gaussian case and the combined Gaussian and impulsive case. On the other hand, Tables 3 and 7 and Tables 4 and 8 clearly indicate the sensitivity of the periodogram method to fluctuations from the original Gaussian noise assumption.

As discussed above, Tables 5 and 6 show that, in the case of the robust method, permutation-based significance value computation performs approximately equally well as the simulation-based computation. The only notable difference is again seen in the number of found periodic genes for short time series (e.g., *N *= 40, 45, 50) and small FDR levels (*q = *0.005, 0.01, 0.05). Tables 7 and 8 in turn show that, apart from the *Z*-scores, the permutation-based method mitigates the sensitivity of periodogram method to the fluctuations from the model (Gaussian) distribution.

The robustness of the proposed method is further demonstrated by its response to combined Gaussian noise and nonlinear cubic distortion. As explained in Computational Methods Section, the robust method depends on the observed time series only through the rank sequence. Any monotone distortion preserves the ordering of the samples. Therefore, the rank-based method is completely insensitive to any monotone distortions. Consequently, the results for the third test case are identical to those presented in Tables 1 and 2. The results for the periodogram method are shown in Table 9.

### Experimental results

The data sets that are considered here are from the following papers: [6,5,7]. For each time series experiment (13 in total), we apply the proposed robust methods for detecting genes having both fixed and unknown frequency components. For the fixed frequency we use the one that corresponds to the length of the cell cycle. Following the idea presented in [8], a simple method for estimating the cell cycle length/frequency is to compute the average robust spectral estimate. For each time series, we present the number of statistically significant genes that are found to be periodically behaving at a specific level of the FDR (*q = *0.05). For the cdc15 experiment by Spellman *et al. *[6], the sampling time was not equidistant in the beginning and at the end of the data set. Considering the missing time points as missing values would result in a large number of missing values with a regular pattern of occurrence. Although the proposed robust methods can cope with missing values, such a regular pattern of missing values can artificially cause many small significance values and hence result in an unreliably large number of statistically significant periodic genes. This can be avoided e.g. by interpolating the expression values for the systematically missing time points, in which we used simple linear interpolation. Only non-missing expression values are considered in the interpolation. If the expression values of both the previous and the next time instants are missing, then the interpolated sample is defined to be missing as well. This results in a more conservative number of detected genes for the cdc15 experiment. We chose to consider only those genes that have less than 30% missing values and decided to rule out all except the Score3 experiment in the data by Whitfield *et al. *[5] because of high degree of irregular sampling and short time series length. The obtained results are shown in Table 10. The total number of genes analysed in each data set is denoted as *M*. The number of found periodic genes having fixed and unknown frequency are denoted as *P' *and *P*, respectively. The corresponding figures from [8] are shown in parentheses. For the detection of periodic components having unknown frequency, we used the permutation-based method. As was shown in Simulations Section, both the simulation and permutation based approaches performed approximately equally well. Hence, for the ease of implementation, we used the simulation-based method for the detection of periodic components having a fixed frequency.

If we first take a look at the numbers of detected genes having a periodic component of an unknown frequency (*P*) shown in Table 10, we can see that generally the numbers of periodically behaving genes are lower than those of the corresponding figures in [8]. In other words, the proposed robust method seems to provide more conservative estimates, although the cdc15 experiments shows an exception. In the case of real data further comparison between the two methods is much more subjective than in simulation experiments since the ground truth is not completely known. Based on the simulation results shown above, one could put more faith on the robust method, especially in the cases where the Gaussian noise assumption is violated. Hence one could argue that the robust method has ignored more non-periodic time series, particularly ones where outliers and other non-idealities have caused artificial variation in the periodogram. The number of detected truly periodic genes can be increased, at the cost of detecting more false positives, by using a higher level of *q*.

Let us then focus on the numbers of found periodic genes when using a fixed frequency in the robust method (*P*'). Concerning the numbers of detected periodic genes in the data sets by Spellman *et al. *[6], the results are in concordance with the previously published ones [24]. On the cdc28 data set, the proposed method finds a slightly higher number of periodic genes. Direct comparison between the numbers *P *and *P' *is not meaningful as the number of detected genes depends on the significance level, which may be dependent on the used method. The comparison is better done using the ordered gene lists which we will discuss shortly together with three benchmark gene sets (see below).

We have not yet come across with another study, besides the original paper, that would have examined the periodicity in the data by Rustici *et al. *[7]. Rustici *et al. *investigate the global cell cycle control of gene expression in the fission yeast *Schizosaccharomyces pombe *using DNA microarrays. Thus, the comparison of values in Table 10, related to Rustici *et al. *data, is not feasible. Table 10 shows that the number of detected periodic genes ranges from 673 up to 3131. In the case of very large number of detected periodic genes, more insight into the data set can be gained by looking at the ordered list of genes.

**Figure 4:**
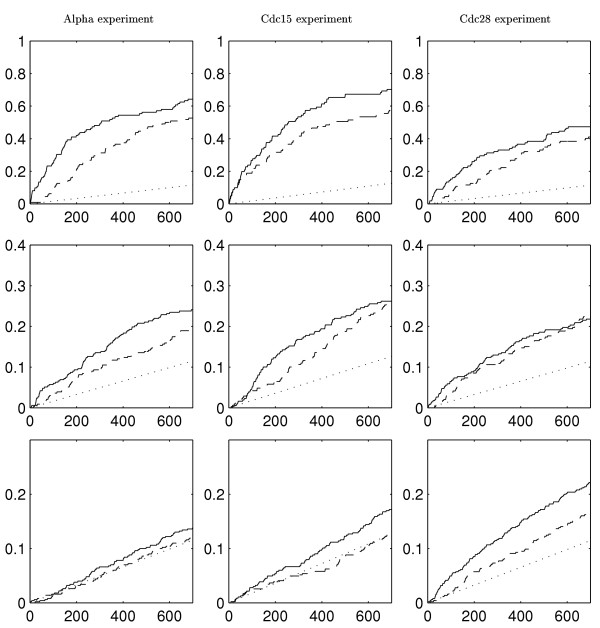
**Benchmark results**. The fraction of the benchmark set that is identified (*y*-axis) as the function of the highest ranked genes (*x*-axis). The solid (resp. dashed) line corresponds the robust detection having fixed (resp. unknown) frequency. The dotted line shows the performance of the random gene selection. The columns from left to right correspond to the Alpha, the Cdcl5 and the Cdc28 experiment by [6]. The rows, from top to bottom, correspond to the three different benchmark gene sets B1, B2 and B3. for more details about the benchmark gene sets, see [24].

Table 10 only shows the number of detected genes. Further insight can be gained by looking at the enrichment of the genes assumed to be cell cycle regulated among the top ranked genes. In particular, we resort to the three different benchmark gene sets introduced in [24]. In order to provide a direct comparison with the results shown in [24], we show similar enrichment graphs for the both robust detection methods (fixed and unknown frequency) in Figure 4. Note that some of the benchmark genes are ignored during the analysis since they have more than 30% missing values. There are also a few benchmark genes for which no exact match was found among the genes in the data provided by [6]. Hence the graphs are drawn based on slightly smaller benchmark gene sets, namely, 101–112 (B1), 309–339 (B2), and 465–505 (B3), depending on the experiment.

Figure 4 reveals some interesting results. First, let us compare how well the proposed robust method (with the fixed frequency) finds the benchmark genes when compared to previously published methods (for comparison, see the corresponding graphs in [24]).

The results for the benchmark gene set B1 are shown on the first row in Figure 4. For all the data sets, the performance of the robust method is either between the best methods and the amplitude-independent method by Zhao *et al. *[9], or close to the method by Zhao *et al. *[9]. This finding is not surprising. As discussed in [24], this benchmark gene set is biased towards periodic genes which are strongly regulated, i.e., have large amplitudes. In general, because high-amplitude periodic genes are more easily detected from noisy expression data, the gene sets identified from such studies are likely to be biased towards high amplitude genes. An advantage of amplitude-independent methods is, however, that they detect small-amplitude periodic transcripts better, and hence may identify genes which are not yet known to be periodic.

For the benchmark gene set B2, the performance of the proposed robust method is approximately the same as that of the best methods reported in [24], except for the alpha experiments on which the robust method performs slightly worse. Noteworthy is that the benchmark gene set B2 is obtained from a separate Chromatin IP experiment and thus is independent of the previous gene expression studies.

Concerning the benchmark gene set B3, the robust method performs better than the majority of the methods. Notably good performance is seen on the data from the Cdcl5 and the Cdc28 experiments. Interestingly, the benchmark set B3 is also likely to be biased, but towards small amplitude genes [24]. This strengthens the assumption that the potential of the proposed robust method is especially in detecting unknown, small-amplitude, periodic genes.

Yet another interesting observation can be drawn by comparing the solid and dashed curves in Figure 4. As can be expected, the method which detects especially cell cycle frequencies ranks the benchmark genes higher than the method which detects unknown (all) frequencies, i.e., the solid line is above the dashed line. Another expected behavior is that the method which detects unknown frequencies also detected a great number of genes assumed to be related to the cell cycle. However, from another point of view, Figure 4 also indicates that there are some statistically significant periodic patterns which are more significant than some of the cell cycle related ones. Possible sources of those significant periodic patterns may include, among others, systematic artifacts in the array/experiment preparation, unknown periodic biological processes, or simply the considerable amount of experimental noise (false positives).

The top 300 ranked genes for all the data sets analysed, obtained using the proposed robust method, are provided on our companion website.

## Discussion

As discussed above, some extensions and improvements over the traditional periodogram/correlogram approach have been proposed in the literature. Two particular modifications, namely utilisation of windowing and a trimmed *g*-statistic, were reported to provide a good performance in a recent review by Artis *et al. *[20]. Although we provided an extensive comparison of the unmodified traditional and the (unmodified) proposed rank-based methodologies, the further modifications can be implemented in a straightforward fashion in both of the frameworks. These extensions for the robust rank-based method will be examined in future studies but let us give an overview of the possible modifications.

As discussed previously, the biased version of the robust correlation estimator can be viewed as a type of weighting or windowing. More generally the windowing is typically incorporated into the computation of the spectral estimate (see, e.g., Equations (6) and (11)). Different windows provide different properties for spectral estimators. For example, the shape of the window can be used to control the smearing and leakage effects whereas the length of the window compromises with the spectral resolution and the variance [25]. In general, the used windows can be chosen from a general class of windows, including, among others, Bartlett, Daniel, and Parzen windows (see, e.g. [16,18,19]). Concerning the detection of hidden periodicities, windowing can be applied much in the same way as it is used in the method by Priestley.

Similarly, the modification by Chiu [21], i.e., the use of a proper trimmed mean of the ranked periodogram ordinates in place of the average periodogram, can be applied to the robust rank-based estimator as well. A drawback associated with the use of the trimmed *g*-statistic in the traditional periodogram setting is that only asymptotic distribution of the test statistic is available. The discrepancy between the true distribution and the asymptotic one can be remarkable in the case of small sample size typical e.g. in gene expression studies. These difficulties can be circumvented by the computer intensive simulation and permutation-based methods explained above.

The proposed method has other possible extensions as well. As a periodically behaving gene may be involved in several different biological processes, its expression pattern may contain several dominant frequencies. In that regard, the testing procedure can be extended to detect several frequency peaks from the spectral estimate. See, e.g., [19,21] for extensions of Fisher's test to that direction.

In cell cycle related studies, a cell population is usually forced to synchrony prior to taking the measurements using an external synchronisation method. The synchronisation is achieved by arresting the cells at a specific phase of the cell cycle after which they are released. However, as time evolves, the cell population gradually loses its synchrony. Such a phenomenon can be viewed as time-varying (low-pass) filtering of the expression values where the time-varying filter kernel corresponds to the distribution of the cell population over the cell cycle. Inverse methods have been developed to correct for the effect of the loss of synchrony [26,27]. Several interesting questions remain to be studied. First, the inverse filtering problem as such is fairly sensitive to noise and is further complicated by the fact that the accuracy level at which the filter kernel (i.e., distribution of the cell population) can be measured is limited. Therefore, the corrected time series may contain even more obscure non-idealities than the uncorrected ones.

Consequently, robust methods are potentially even more important when periodic components are sought from the time series which are corrected for the loss of synchrony. Future studies are also needed to compare the robust periodicity detection method, when applied to both uncorrected and corrected time series, to see whether the inversion of the loss of synchrony brings any additional gain in the case of robust periodicity detection. In addition to the simulation results presented in the Simulations Section, we also performed preliminary simulations where the amplitude of the periodic signal was attenuated to model the loss of synchronisation. We noticed that if the average amplitude of the sinusoidal signal remained the same, the results were similar to those in the tables of the Simulations Section.

Further comparisons must also be made to assess the performance differences between the proposed method (possibly combined with a proper inversion method for the loss of synchrony) and alternative methods in which a model for the loss of synchrony is incorporated into the statistical testing framework [12,11]. Although elegant, such combined approaches have potential difficulties in that they usually result in a computationally intensive optimisation problem [11] and/or include several distributional assumptions [12]. Furthermore, the inversion of the loss of synchrony is performed blindly, i.e., without any additional measurements, which the distribution of the cell population could be estimated with. Future experiments are needed to address these questions.

## Conclusion

The presented method yields a robust way of finding periodicity in short time series data. As illustrated in Simulations Section, the proposed robust detection method is remarkably insensitive to different kinds of non-idealities in the data, such as heavy contamination of outliers, missing values, short time series, nonlinear distortions, and is completely insensitive to any monotone nonlinear distortions. The results also show that the proposed method has clearly better performance than the Fisher's test, even in the case of the standard Gaussian noise. Furthermore, the results on real data demonstrate that the proposed method performs well on real data and that the results are biologically meaningful. As illustrated in Figures 2(a)–(c) and more extensively reported in [14], the robust method serves also as a good spectral estimator. As the time series measured from biological systems are usually short and prone to contain different kinds of non-idealities, we believe that the robust detection method presented in this paper will find many important applications in this field.

## Authors' contributions

MA carried out an implementation of the methods, performed the computations and co-drafted the manuscript. HL developed the statistical methods, helped in computations and mainly drafted the manuscript. RP helped in developing the statistical methods. OY-H conceived of the study and participated in its design and coordination. HH helped in the implementation of the computational methods. All authors read and approved the final manuscript.

## Figures and Tables

**Table 1:** Number of inferred periodic time series: case 1. The number of inferred periodic time series using the robust method and standard Gaussian noise in the data. *p*-values were obtained by simulating the distribution of the *g*-statistic using 10000 time series composed of Gaussian noise.

*q\N*	10	20	40	45	50	100
0.15	0	2(1)	107(90)	109(96)	117(99)	115(100)
0.10	0	1(1)	96(87)	103(94)	110(98)	109(100)
0.05	0	1(0)	83(79)	95(89)	101(96)	105(100)
0.01	0	1(0)	59(59)	80(79)	90(89)	101(100)
0.005	0	0(0)	32(32)	62(61)	64(64)	100(100)
						
*Z*	12	49	89	93	95	100

**Table 2:** Number of inferred periodic time series: case 2. The number of inferred periodic time series using the robust method and standard Gaussian noise in the data. *p*-values were obtained by using permutation tests.

*q\N*	10	20	40	45	50	100
0.15	0	4(3)	108(92)	113(96)	111(98)	119(100)
0.10	0	1(1)	99(90)	106(94)	106(97)	112(100)
0.05	0	1(0)	88(84)	97(89)	101(95)	106(100)
0.01	0	0	65(64)	80(78)	86(86)	101(100)
0.005	0	0	46(46)	61(61)	71(71)	100(100)
						
*Z*	15	48	91	92	95	100

**Table 3:** Number of inferred periodic time series: case 3. The number of inferred periodic time series using the periodogram method and standard Gaussian noise in the data. *p*-values were obtained by using Equation 5.

*q\N*	10	20	40	45	50	100
0.15	1(0)	17(11)	111(89)	108(86)	117(97)	122(100)
0.10	0	9(6)	100(85)	98(80)	108(96)	115(100)
0.05	0	2(1)	83(78)	79(71)	99(93)	107(100)
0.01	0	0	56(55)	54(52)	84(83)	101(100)
0.005	0	0	23(23)	20(20)	54(54)	99(99)
						
*Z*	13	39	85	83	93	99

**Table 4:** Number of inferred periodic time series: case 4. The number of inferred periodic time series using the periodogram method and standard Gaussian noise in the data. *p*-values were obtained by using permutation tests.

*q\N*	10	20	40	45	50	100
0.15	0	2(0)	109(94)	111(98)	114(98)	117(100)
0.10	0	2(0)	98(91)	103(96)	107(98)	112(100)
0.05	0	2(0)	87(84)	98(95)	103(98)	105(100)
0.01	0	2(0)	68(67)	88(86)	91(80)	102(100)
0.005	0	1(0)	55(55)	70(70)	80(79)	100(100)
						
*Z*	14	40	92	94	97	100

**Table 5:** Number of inferred periodic time series: case 5. The number of inferred periodic time series using the robust method and standard Gaussian plus impulsive noise in the data. *p*-values were obtained by simulating the distribution of the *g*-statistic using 10000 time series composed of Gaussian noise.

*q\N*	10	20	40	45	50	100
0.15	0	1(0)	73(62)	84(73)	101(84)	114(100)
0.10	0	1(0)	60(55)	76(69)	91(81)	109(100)
0.05	0	1(0)	48(46)	58(56)	77(73)	105(100)
0.01	0	0	22(21)	21(21)	49(49)	100(99)
0.005	0	0	13(13)	17(17)	34(34)	95(95)
						
*Z*	9	35	73	78	84	99

**Table 6:** Number of inferred periodic time series: case 6. The number of inferred periodic time series using the robust method and standard Gaussian plus impulsive noise in the data. *p*-values were obtained by using permutation tests.

*q\N*	10	20	40	45	50	100
0.15	0	1(0)	70(57)	83(72)	93(82)	117(100)
0.10	0	0	59(51)	71(68)	89(79)	111(100)
0.05	0	0	48(45)	60(58)	77(72)	105(99)
0.01	0	0	24(23)	35(33)	54(53)	99(98)
0.005	0	0	22(21)	27(27)	41(41)	95(95)
						
*Z*	9	38	72	78	82	99

**Table 7:** Number of inferred periodic time series: case 7. The number of inferred periodic time series using the periodogram method and standard Gaussian plus impulsive noise in the data. *p*-values were obtained by using Equation 5.

*q\N*	10	20	40	45	50	100
0.15	0	0	16(16)	24(22)	28(28)	77(71)
0.10	0	0	13(12)	20(12)	25(24)	73(69)
0.05	0	0	11(11)	13(13)	17(17)	65(63)
0.01	0	0	3(3)	6(6)	9(9)	52(52)
0.005	0	0	0(0)	1(1)	3(3)	40(40)
						
*Z*	11	29	44	46	52	79

**Table 8:** Number of inferred periodic time series: case 8. The number of inferred periodic time series using the periodogram method and standard Gaussian plus impulsive noise in the data. *p*-values were obtained by using permutation tests.

*q\N*	10	20	40	45	50	100
0.15	0	6(5)	38(29)	40(33)	18(16)	82(76)
0.10	0	2(2)	34(26)	36(32)	14(13)	76(72)
0.05	0	1(1)	23(22)	32(29)	10(9)	69(69)
0.01	0	0	16(15)	18(18)	7(6)	61(61)
0.005	0	0	15(14)	17(17)	7(6)	53(53)
						
*Z*	11	32	47	49	44	80

**Table 9:** Number of inferred periodic time series: case 9. The number of inferred periodic time series using the periodogram method and standard Gaussian noise and cubic distortion in the data. *p*-values were obtained by using permutation tests.

*q\N*	10	20	40	45	50	100
0.15	0	0	49(44)	79(64)	89(74)	107(93)
0.10	0	0	39(36)	71(62)	80(69)	98(90)
0.05	0	0	25(24)	52(49)	64(59)	90(88)
0.01	0	0	8(8)	28(28)	44(43)	82(82)
0.005	0	0	8(8)	19(19)	37(36)	67(67)
						
*Z*	7	15	68	71	79	91

**Table 10:** Number of inferred periodic genes from real microarray data. Results obtained by using real microarray data are presented here. Permutation tests were used to obtain significance values for the robust spectra. Symbols: *N *is the length of the time series, *M *is the number of genes analysed, *P *(resp. *P*') is the number of found periodic genes having an unknown frequency (resp. frequency corresponding to the cell cycle length). Notes in the table: ^a^Since the elutriation time course did not show any significant periodic components, we did not perform the test with a fixed frequency. ^b^The average spectrum showed several major peaks. The one in the vicinity of the assumed cell cycle frequency was chosen manually.

Cell type	Experiment	*N*	M	*P'*	*P'/M*	*P*	*P/M*	Source
S.cerevisiae	cdc15	24	5287	981	18.6	946(766)	17.9	[6]
S.cerevisiae	cdc28	17	6103	363	6.0	32(105)	0.5	
S.cerevisiae	alpha	18	6056	346	5.7	139(468)	2.3	
S.cerevisiae	elutriation	14	6074	_a	_a	4(193)	0.07	
Human HeLa	Score3	48	41508	1285^b^	3.1^*b*^	3580(6043)	9.0	[5]
S.pombe	cdc25-1	19	4373	1431	32.7	759	17.4	[7]
S.pombe	cdc25-2	36	4422	2605	58.9	2197	49.7	
S.pombe	cdc25-sep1	20	4700	2624	55.8	2295	48.8	
S.pombe	elutriation1	20	4229	1948	46.1	551	13.0	
S.pombe	elutriation2	20	3961	1453	36.7	384	9.7	
S.pombe	elutriation3	20	4236	673	15.9	355	8.4	
S.pombe	elutr.-cdc10-br	22	4647	3131	67.4	2431	52.3	
S.pombe	elutr.-cdc25-br	21	4272	2405	56.3	767	18.0	
